# Perforated Calculous Cholecystitis and Incidental Squamous Cell Carcinoma of the Gallbladder—A Complex Relationship with a Difficult Management in the Acute Setting

**DOI:** 10.3390/medicina61030452

**Published:** 2025-03-05

**Authors:** Matteo Zanchetta, Gian Luigi Adani, Giorgio Micheletti, Gianmario Edoardo Poto, Stefania Angela Piccioni, Ludovico Carbone, Ilaria Monteleone, Marta Sandini, Daniele Marrelli, Natale Calomino

**Affiliations:** 1 Unit of General Surgery and Surgical Oncology, Department of Medicine, Surgery and Neurosciences, University of Siena, Viale Mario Bracci 16, 53100 Siena, Italy; 2 Kidney Transplant Unit, Department of Medicine, Surgery and Neuroscience, Siena University Hospital, University of Siena, Viale Mario Bracci 16, 53100 Siena, Italy; 3 Diagnostic Imaging Unit, Department of Medical, Surgical and Neurosciences, Siena University Hospital, Azienda Ospedaliera Universitaria Senese, Viale Bracci 10, 53100 Siena, Italy

**Keywords:** cholelithiasis, gallbladder carcinoma, squamous cell carcinoma, diabetes mellitus, gallstones, peritonitis, acute cholecystitis, tissue hypoxia, emergency surgery, carcinogenic microenvironment

## Abstract

The worldwide prevalence of gallstones (GSs) is estimated to be between 10% and 15% in the general population. Gallbladder carcinoma (GBC) is the most common biliary tract neoplasia, and it is characterized by highly aggressive behavior and poor overall prognosis. Long-standing GSs and chronic inflammatory state represent the most common risk factors for GBC, promoting a carcinogenic microenvironment. Long-standing GSs expose patients to potentially severe surgical and oncological complications. A 71-year-old gentleman, who had never experienced biliary symptoms and had diabetes mellitus (DM), presented with severe peritonitis due to perforated acute calculous cholecystitis. The patient underwent an emergent laparotomic cholecystectomy. Histopathology found a rare pT2b poorly differentiated squamocellular carcinoma of the gallbladder. Although more difficult due to the concomitant inflammatory context, it is critical to identify suspicious lesions during preoperative imaging in patients at high risk of malignancy presenting with complex acute gallbladder pathologies. A review of the literature was conducted to gain a deeper insight into the relationship between long-standing GSs and GBC, evaluating also the difficult diagnosis and management of malignancy in the acute setting. Considering the existing literature, the choice to pursue a prophylactic cholecystectomy may be justifiable in selected asymptomatic GS patients at high risk for GBC.

## 1. Introduction

The worldwide prevalence of gallstones (GSs) is estimated to be between 10% and 15% in the general population [1]. Some known risk factors for cholelithiasis development are obesity, female gender, increasing age, rapid weight loss, and a sedentary lifestyle, in association with metabolic disorders and genetic predisposition [2,3]. As 20% to 40% of patients with cholelithiasis will develop complications during their lifetime, acute cholecystitis is the first clinical presentation in 10–15% [1]. Gallbladder perforation and peritonitis are rare but potentially life-threatening complications of cholelithiasis [4]. Gallbladder carcinoma (GBC) is the most common biliary tract cancer, characterized by highly aggressive behavior and poor overall prognosis [5]. Long-standing cholelithiasis and a chronic inflammatory state represent the most common risk factors for GBC [6,7], and some potential risk factors for its development in patients with GS have been proposed [8]. The transition from healthy tissues subjected to chronic inflammatory noxae to carcinoma involves multiple molecular pathways, including many associated with hypoxic conditions such as hypoxia-inducible factors (HIF), C-X-C motif chemokine receptor 4 (CXCR4), and others [9,10]. Diabetes mellitus (DM) is a significant contributing factor to the increased risk of GBC, simultaneously promoting the development of GSs and creating a carcinogenic microenvironment [11,12]. A brief review of the existing literature was conducted with the objective of gaining a deeper insight and understanding of the multifaceted relationship between long-standing cholelithiasis and the development of GBC, including the intervening role of DM, the difficult diagnosis of malignancy in the context of acute cholecystitis, and an examination of the role of prophylactic cholecystectomy in highly selected asymptomatic patients, which has not been unanimously agreed upon.

## 2. Case Report

**Figure 1:**
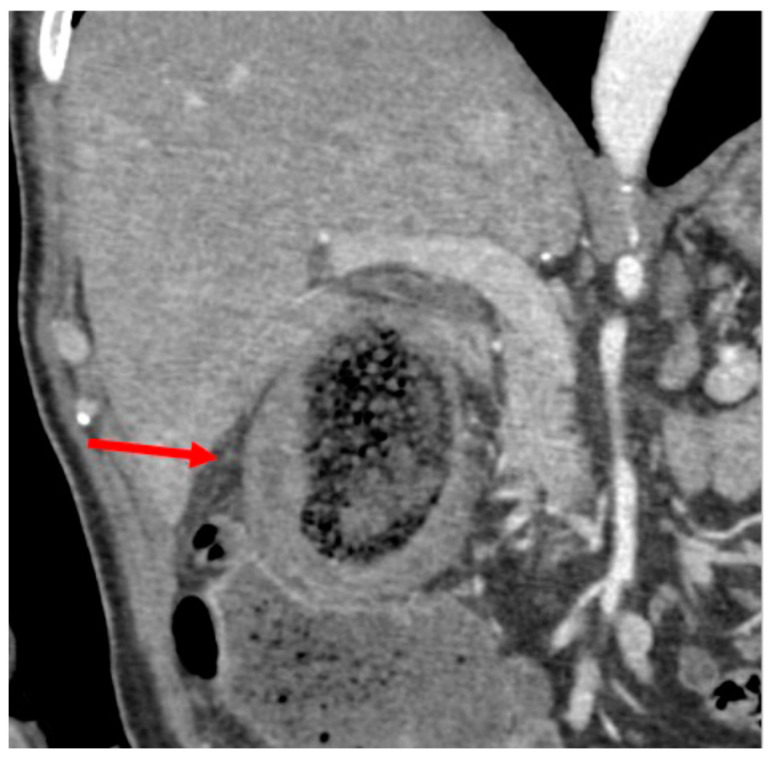
CT scan: thickened gallbladder walls with loss of normal wall stratification (arrow).

A 71-year-old gentleman presented to the Emergency Department (ED) of our hospital with acute abdominal pain (Numeric Pain Rating Scale 8–9) rapidly developed over the afternoon, associated with fever (body temperature 38.8 °C), nausea and vomiting, and bowel closed to gas and feces. On physical examination, the abdomen was tender with guarding, Blumberg sign was strongly positive in the right hypochondrium and right flank, and Murphy sign was notably present. Laboratory tests showed elevation of leukocytes (11.3 × 10^9^/L), procalcitonin (7 μg/L), and C-reactive protein (25 mg/dL). He denied any past biliary symptoms or abdominal discomfort of any sort and was not aware of having cholelithiasis. A review of the patient’s medical history revealed DM, and adenoidectomy in pediatric age. Given the severity of the clinical picture, an abdominal computed tomography (CT) scan was ordered by the on-duty surgeon. The gallbladder was markedly distended due to the presence of intraluminal GSs, with minimal dilation of the intrahepatic bile ducts. The gallbladder walls exhibited diffuse thickening, accompanied by a loss of the physiological parietal stratification, as discernible in all post-contrast phases (Figure 1).

**Figure 2:**
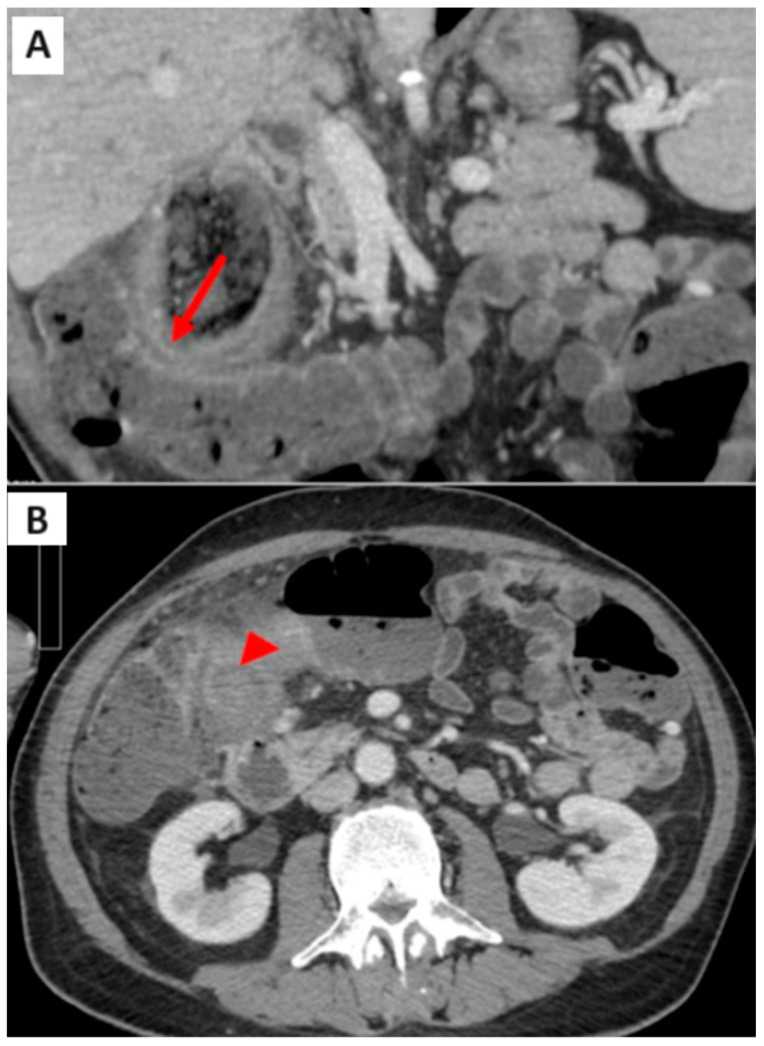
(**A**) CT scan: proximity of gallbladder wall, pericholecystic collection, and right colonic flexure (arrow); (**B**) CT scan: fat stranding of the pericholecystic tissue (arrowhead).

Additionally, a defect was present in the inferomedial wall of the gallbladder, draining into a pericholecystic collection, predominantly gas-filled, in close proximity to the right flexure of the colon. Here, colonic walls appeared hypervascularized, suggesting an inflammatory condition (Figure 2A). Another noteworthy finding was the extensive fat stranding of the pericholecystic adipose tissue, which was more evident in the late phases acquired using dual-energy imaging (Figure 2B).

**Figure 3:**
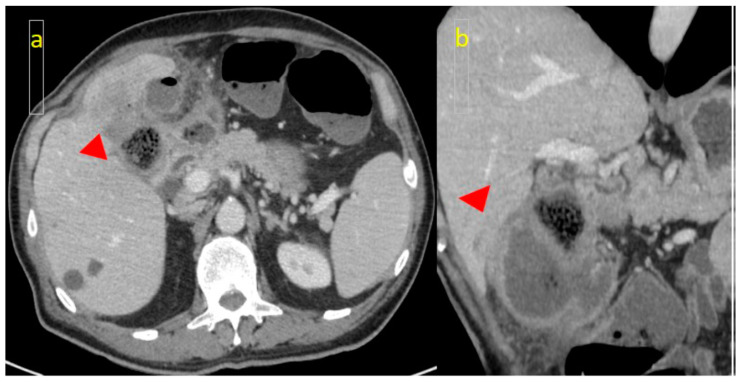
(**a**) CT scan: hypodense area (arrowhead) of the pericholecystic liver parenchyma, suggestive of invasion; (**b**) Coronal CT slice.

Furthermore, in all post-contrast phases, as well as in double energy, a hypodense area was present in the pericholecystic liver parenchyma (Figure 3a,b).

**Figure 4:**
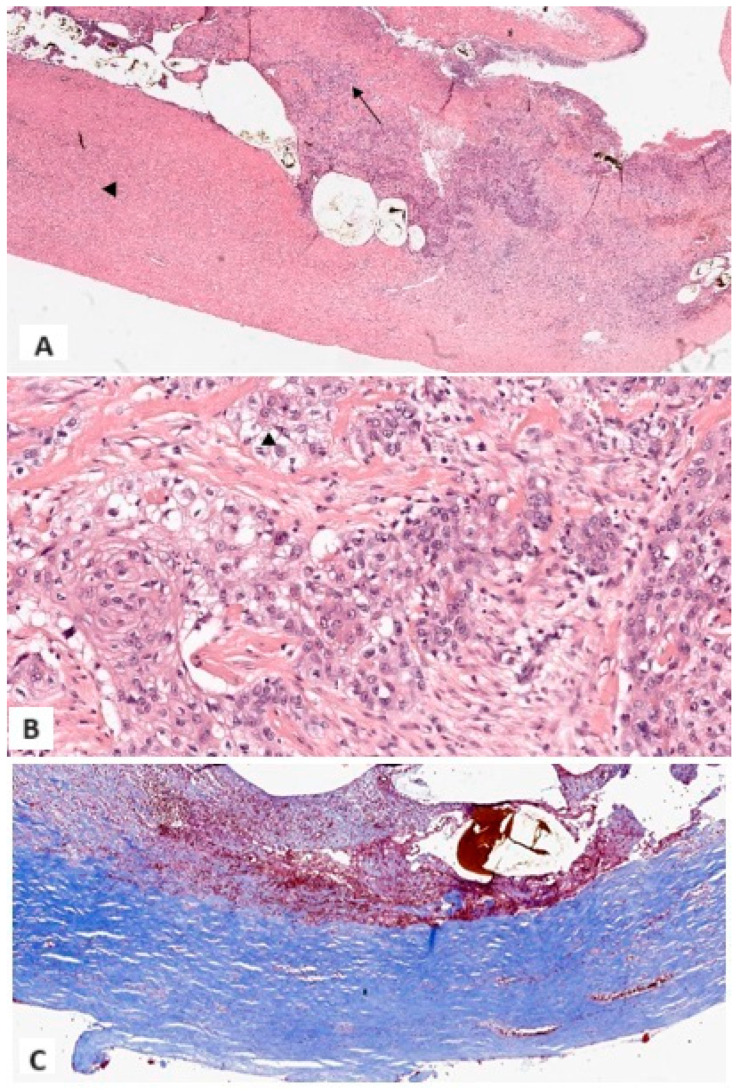
(**A**) At lower magnification, xanthocholegranulomatous inflammation (arrow), lamellar hyaline fibrosis (arrowhead), ulceration, and fibrin deposits, with widespread loss of epithelium (1×, Hematoxylin and Eosin); (**B**) Malignant squamous cells separated by dense and scattered fibrosis. Aspects with clear cytoplasm were documented (13×, Hematoxylin and Eosin); (**C**) Dense lamellar fibrosis with loss of the fibromuscular layer. No dystrophic calcifications were identified (6×, Masson Trichrome Stain).

The surgeon performed an emergency explorative laparotomy with subsequent cholecystectomy and resection of the gallbladder hepatic bed. The gallbladder was perforated, and numerous GSs were already dispersed within the peritoneal cavity, with only few remaining within the gallbladder itself. The intraoperative examination of the gallbladder wall revealed a specific area of increased thickness and firmness when compared to the surrounding inflamed and thickened wall. This area was identified as being located at a different site from the perforation, and the intraoperative examination already indicated a high degree of suspicion for malignancy. Macroscopically, the gallbladder measured 7 cm × 3.5 cm and had a firm and rigid wall with a thickness ranging from 0.5 to 2 cm. The serosal surface was brown, hemorrhagic, and irregular. The mucosal surface was ulcerated, and heterogeneous in thickness and color (pale tan and brown). A wide full-thickness parietal break was present, distant from the bulk of the malignant lesion. Microscopically, the entire gallbladder wall exhibited diffuse paucicellular lamellar hyaline fibrosis and sclerosis, with complete loss of the muscular layer. In the thickest areas, scattered multiple foci of chronic xanthocholegranulomatous inflammation were identified, mainly localized around intraparietal stones. A pT2b poorly differentiated carcinoma of the gallbladder with squamocellular (SCC) differentiation was identified [immunophenotype: high molecular weight cytokeratin + (CKHMW), AE1-AE3 +, epithelial membrane antigen + (EMA), cytokeratin 7 − (CK7), synaptophysin-], distant from the site of perforation. The epithelial neoplastic elements were arranged in a variety of patterns, including single cells, cords, islands, and sheets, and were found to be intermingled with a dense fibrous stroma with inflammatory infiltrates (Figure 4A–C). The specimen obtained during the emergency surgical procedure included, in addition to the gallbladder itself, a 5 cm × 4 cm piece of hepatic parenchyma, corresponding to an area slightly larger than the gallbladder bed, which was not infiltrated by carcinomatous cells.

The immediate post-operative course was unremarkable. Given the intraoperative suspicion of neoplasia, serum markers were measured after surgery, yielding values for cancer antigen 19-9 (CA 19-9), cancer antigen 125 (CA 125), and carcinoembryonic antigen (CEA) of 70.1 U/mL, 30.9 U/mL, and 1.3 ng/mL, respectively. The patient was referred to the oncological follow-up (FU). A staging CT scan was performed approximately one month after the first surgical procedure, showing diffuse inflammation of the tissues surrounding the hepatic bed of the gallbladder, with fat stranding, several peritoneal micronodules, especially along the right paracolic groove (potentially attributable to residual GS elements that spread after the cholecystic perforation), enlarged lymph nodes around the hepatic hilum (max 8.5 mm), and a moderate amount of ascitic fluid. One month later, once the inflammation had reduced, an abdominal explorative laparoscopy was conducted. Several of the aforementioned micronodules were identified on both the parietal and visceral peritoneum. During the procedure, many biopsies were taken, including many of the identified micronodules (both in the supra-mesocolic and sub-mesocolic region), and intraabdominal fluid samples for cytological examination were collected. No evidence of peritoneal carcinomatosis was identified, although a lymph node from abdominal station 8a (common hepatic artery nodes) exhibited carcinomatous infiltration, demonstrating a positive reaction with anti-cytokeratin antibodies AE1 and AE3 (as the primary tumor lesion in the gallbladder wall). The cytologic examination was negative. Following a collegial discussion, the decision was taken to commence adjuvant chemotherapy comprising a combination of cisplatin and gemcitabine. The tumor markers CEA and CA 19-9 exhibited levels of 2.4 ng/mL and 96.5 U/mL, respectively, six months following the initial surgical procedure. At the nine-month mark, these levels increased to 2.6 ng/mL and 112.0 U/mL, respectively. A CT evaluation performed nine months after the index surgery revealed the development of three small nodular lesions in the right subdiaphragmatic region external to segment IV of the liver (progressively growing from 10 to 16 mm), in the omentum anteriorly to the hepatic flexure of the colon (stable over time, smaller than 10 mm), and in the pelvic peritoneal rectovesical excavation (progressively growing but still smaller than 10 mm), respectively. A subsequent CT scan evaluation performed fifteen months later revealed no evidence of progression or emergence of new peritoneal localizations. Fifteen months after the initial surgical procedure, the patient’s overall health status remains satisfactory at the FU.

## 3. Discussion

Acute calculous cholecystitis accounts for a large proportion of emergency surgical admissions to the ED, as it may lead to severe complications if not promptly treated [1,13]. Risk factors associated with the development of GSs include obesity, female gender, increasing age, rapid weight loss, a sedentary lifestyle, and DM and hyperlipidemia [2,3,14]. Intraluminal GSs are inherently detrimental to the surrounding tissues, given their propensity to elicit an inflammatory response over time. This persistent inflammation may eventually degenerate in complications such as secondary bacterial infection and, in severe cases, ischemia, necrosis, and life-threatening perforation of the gallbladder wall [15]. The latter is observed in 2% to 11% of acute events [16]. Gallbladder carcinoma is a malignant tumor of the biliary tract, highly aggressive but relatively uncommon [5], estimated to account for approximately 1.2% (about 220,000 patients in 2018) of all cancer diagnoses but 1.7% (about 165,000 patients) of cancer-related deaths worldwide [17]. The prevalence shows significant geographic variation, with notably higher rates in certain regions, such as South America (27 per 100,000 persons), India [18], and East Asia, and lower in other ones, such as North America (1.5 per 100,000 persons) [19]. The average age at diagnosis is 71 years, with women affected two to six times more frequently than men [20]. Gallbladder cancer is a particularly lethal disease due to its nonspecific symptomatology and subsequent tendency to be diagnosed at an advanced stage, when treatment options are limited [21]. Indeed, only a minority of GBC patients are diagnosed at an early stage, when the prognosis might still be favorable, also due to the lack of specific tumor biomarkers and tendency to be asymptomatic until later stages [22]. In the United States, only approximately 20% of GBCs are diagnosed in the early stages [23]. The 5-year survival rate is estimated to be 60% for stage I, i.e., confined to the gallbladder, and less than 5% for infiltrative stage III or IV tumors [7,24]. One of the cardinal factors in the genesis of GBC, as in a multitude of other solid tumors, seems to be the perpetuation of a state of inflammation [25]. This concept is based also on evidence that the most common risk factors for the development of GBC are long-standing GSs and chronic cholecystitis (Table 1) [26,27,28]. Other major etiological factors reported are advanced age, female sex, certain genetic variants, obesity, DM, occupational exposure to carcinogens, chronic biliary infections, and high parity [17,29,30]. It is noteworthy that female gender, multiparity, DM, and obesity are major risk factors also for cholesterol GS formation. Porcelain gallbladder and atrophic gallbladder are considered high-risk factors for GBC, and approximately 90–95% of cases of porcelain gallbladder are associated with GSs [31,32,33].

While the overall incidence of GBC in GS patients reportedly ranges from 0.5% to 1.5% [22,34], the presence of cholelithiasis has been reported in 70% to 95% of GBC patients [35,36]. The length of time that GSs are retained within the gallbladder appears to be a critical factor in determining the nature of this association. Individuals diagnosed with GSs are at a significantly elevated risk of developing GBC, with up to a sevenfold higher incidence, or more, compared to the general population [37]. The size of the GS has been identified as a contributing factor in the severity of the risk of gallbladder carcinoma. Gallstones measuring greater than 2–3 cm have been found to carry a higher risk of malignancy, about 10 times higher than smaller (less than 1 cm) size stones [27,37,38,39]. Similarly, the greater the number of stones, as in the case of our patient, the greater the association with GBC [39,40,41,42]. The elements composing the GSs may also be important, with cholesterol stones showing the greatest risk [21,37].

**Table 1:** Relationship between cholelithiasis and gallbladder carcinoma (chronological order).

Author	Year	Conclusions
Diehl et al. [39]	1983	GSs are a major risk factor for GBC. Large GSs increase the risk for GBC. With stone diameters 2.0–2.9 cm, the odds ratio (vs. stone < 1 cm) was 2.4; for stones 3 cm or larger, the ratio was 10.1.
Csendes et al. [41]	2000	In the asymptomatic GS group, there were significantly more patients with one stone, whereas in GBC patients, there were significantly more with multiple GSs. Patients with GBC had significantly larger GSs.
Serra et al. [40]	2002	The association between GSs and GBC is mediated by the length of time that the stones remain in the lumen of the gallbladder. Longer times permit chronic trauma to the mucosa, initiating a sequence of pathologic changes that can result in GBC.
Misra et al. [27]	2003	GS is an important risk factor for GBC and the epidemiological features of these two diseases are closely linked.
Wistuba et al. [28]	2004	GSs and chronic cholecystitis are important risk factors for GBC.
Roa et al. [42]	2006	GSs are considered the most important risk factor for GBC. Our data suggest that GS weight and volume are significantly higher in GBC than in matched controls. With GS volumes over 10 mL, the relative risk increases by 11 times.
Kapoor [38]	2006	GBC associates with GSs—incidence rates of GBC parallel GS prevalence rates. The role of prophylactic cholecystectomy in patients with asymptomatic GSs to prevent potential malignancies is controversial.
Hsing et al. [35]	2007	GSs are an important risk factor for all three subsites (gallbladder, extrahepatic bile ducts, and ampulla of Vater) of biliary cancer, particularly when complicated by chronic cholecystitis.
Miller et al. [43]	2008	The most important risk factor for GBC is cholelithiasis.
Cariati et al. [36]	2014	Gallbladder carcinoma is related to large and longstanding cholesterol or composite GSs. In our study, GSs were found in 72 of 75 GBC patients.
Hundal et al. [21]	2014	GS is an important risk factor for GBC, being present in almost ~85% of the cases. Further, GBC rates correlate well with the prevalence of GS disease. Groups with high GBC incidence also have a high prevalence of cholesterol GSs.
Jain et al. [25]	2014	Patients with GSs had a high frequency of preneoplastic lesions and accumulation of loss of heterozygosity at various tumor suppressor genes, suggesting a possible causal association of GSs with GBC.
Kanthan et al. [26]	2015	Gallstones cause chronic inflammation that eventually may result in tissue dysplasia. Considering the association between chronic cholecystitis and GBC, prophylactic cholecystectomy may be effective in preventing malignant degeneration.
Sharma et al. [37]	2016	The number of GBC is higher in populations with a high rate of GSs, especially containing higher concentration of organic components (cholesterol), and relatively larger-sized stones.
Calomino et al. [32]	2021	The chronic stimulation by GSs can generate an initial dysplasia that will subsequently turn into GBC.
Lam et al. [33]	2021	Chronic cholecystitis is invariably associated with cholelithiasis, and they share the same risk factors. Complications of chronic cholecystitis include GBC: up to 5% of cases of chronic cholecystitis have been found to have premalignant metaplastic lesions.
Zhu et al. [8]	2023	Age (≤58.5 vs. >58.5 years), size of GS (≤1.95 vs. >1.95 cm), course of GS (≤10 vs. >10 years), CEA (≤5 vs. >5 ng/mL), and CA 19-9 (≤37 vs. >37 U/mL) are independent risk factors for GBC in patients with GSs. When positive indicators were ≥2 among the five independent risk factors or the score of the nomogram was >82.64, the risk of GBC was high in gallstone patients.

Legend. GBC: gallbladder carcinoma. GS: gallstone.

Recently, Zhu et al. proposed five independent risk factors for GBC development in patients with GSs, namely age > 58.5 years, GS size over >1.95 cm, GS presence for over 10 years, serum CEA > 5 ng/mL and CA 19-9 > 37 U/mL [8], stating that the presence of two or more greatly increases the risk of malignancy. The prevailing clinical practice does not advocate prophylactic cholecystectomy in asymptomatic cholelithiasis patients, as surgical intervention is only advised when symptoms or complications have manifested. Considering the strong correlation between long-standing cholelithiasis and the development of GBC, it could be suggested that prophylactic cholecystectomy in certain patients may be a valid option to consider. Some authors have indeed postulated that it should be considered for selected patients at high risk of GBC or other complications (Table 2). We believe that the sentence “There is no innocent gallstone”, which dates back more than a century, might still be nowadays true [44]. In 2005, Kao et al. reached the conclusion that performing a prophylactic cholecystectomy in asymptomatic cholelithiasis patients who had previously undergone a heart transplant appeared to reduce the risk of mortality related to the biliary tract and provided benefits to this population that outweighed the financial costs [45]. Mohandas et al. proposed that prophylactic cholecystectomy should be offered to young healthy women from regions of India at high risk for GBC whenever they are diagnosed to have asymptomatic GSs [46]. Analogously reflecting on the severe burden of GBC in certain areas of India, Mathur suggested that a strategy of prophylactic cholecystectomy in certain patients with asymptomatic cholelithiasis may be beneficial in spite of the surgical risk of the procedures [18]. According to Behari et al., the management for asymptomatic GSs should be selective cholecystectomy in high-risk subgroups, namely patients with associated common bile duct stones, with chronic hemolytic syndromes (e.g., sickle cell disease), or patients with known asymptomatic cholelithiasis who undergo another unrelated abdominal surgery as long as the cholecystectomy does not add to the surgical risk [47]. Illige et al. listed some indications for consideration of prophylactic cholecystectomy in patients with asymptomatic cholelithiasis, namely conditions increasing the risk for GBC (adenomas of the gallbladder, anomalies of the junction between pancreas and the biliary tree, porcelain gallbladder, and solitary gallbladder polyps > 1 cm), and other conditions increasing the risk for the development of related complications (choledocholithiasis, GSs larger than 3 cm, patient living in remote location far from healthcare facilities, sickle cell disease/spherocytosis, transplant or immunosuppressant therapy, and young age) [48]. Similarly, Muroni et al. proposed that prophylactic cholecystectomy should be considered for sickle cell disease patients with asymptomatic cholelithiasis, as it may prove beneficial when considering the potential long-term risk of complications in maintaining the GSs [49]. Recently, Lee et al. performed a Markov decision tree analysis to evaluate the potential benefits of cholecystectomy for asymptomatic GSs, concluding that the option of prophylactic cholecystectomy should always be discussed with patients [50]. They concluded that early prophylactic cholecystectomy could reduce some risk burden especially in selected patients, including individuals with solitary large stones (>1–3 cm) or multiple small stones (<1) that would only increase in size and number over time [51]; organ transplant recipient; those with red blood cell abnormalities (e.g., sickle cell anemia), concurrent gallbladder polyps, or biliary sludge occupying more than 50% of the gallbladder volume; or old but relatively fit patients with numerous GSs in order to avoid anesthetic risks associated with further aging. It is evident that the clinical outcomes for patients with complicated GS disease or, in the most severe cases, GBC, are inferior to those reported for patients undergoing elective laparoscopic cholecystectomy for the treatment of symptomatic biliary colic [52,53]. Liu et al. proposed that prophylactic cholecystectomy should be given due consideration in patients undergoing surgery for gastric cancer, and that it may be performed even during the index oncological procedure [54]. Alves et al. recently concluded that prophylactic cholecystectomy in asymptomatic cholelithiasis is worth considering in heart transplant patients, as there are fewer proportional deaths in those who undergo prophylactic cholecystectomy after transplantation compared to conservative FU and prophylactic cholecystectomy before transplantation, and in patients with biliary microlithiasis [55]. Additionally, they pointed out that whenever GSs and gallbladder polyps coexist, cholecystectomy is indicated in the presence of indirect signs of malignancy related to these polyps, namely thickened, irregular gallbladder wall, increased polyp size during FU, and polyps > 1 cm. Fujita et al. stated that prophylactic cholecystectomy should be considered only for patients at high risk of GBC, such as those with GSs > 3 cm, polyps > 10 mm, porcelain gallbladder, thickened walls of the gallbladder, and stone-filled gallbladders [31]. Considering the risk factors proposed by Zhu et al., our patient was older than the identified age threshold, his blood exams indicated a CA 19-9 level of 70.1 U/mL, some of the numerous GS were estimated to be approximately up to 2 cm according to the operating surgeon, and he most likely had had the stones for an extended period of time. It appears relevant that the patient suffered from DM. Diabetes mellitus has been identified as an important risk factor for the development of GSs [56,57]. Persistent hyperglycemia impairs gallbladder motility, leading to cholestasis and, subsequently, chronic inflammation and oxidative damage within the gallbladder [58,59]. The available literature increasingly indicates DM as a risk factor per se for the development of GBC [12,60,61], and the synchronous presence of GSs has a synergistic effect in increasing the risk of malignancy [62,63]. Hyperglycemia may act as a direct catalyst for tumor growth, directly damaging cellular DNA by altering the expression of oncogenes and tumor suppressors, and providing a higher glucose availability as an energy substrate for fast-growing and highly proliferative cancer cells [64,65,66]. Furthermore, evidence indicates that hyperglycemia is associated with a hypoxic microenvironment, exerting its effects at the cellular level and through chronic vascular alterations [67,68]. The presence of DM in patients undergoing cholecystectomy for acute cholecystitis is associated with an elevated risk of mortality, cardiovascular events, and renal failure [69]. According to a recent meta-analysis, diabetes patients appear to have a higher mortality of GBC compared to non-diabetes patients [70]. The support for prophylactic cholecystectomy could be countered by highlighting the significant financial implications of conducting thousands of prophylactic procedures in asymptomatic GS patients, whilst also acknowledging the 0.5% to 6% complication rate associated with the procedure itself, including potentially severe ones such as common bile duct injury and damage to major blood vessels, along with the ensuing healthcare costs [71,72]. It is theorized that it would be possible to prevent one GBC through the performance of only 67 prophylactic cholecystectomies in areas considered to be high risk, as opposed to 769 cholecystectomies in low-risk areas [18]. Given that Chile has one of the world’s highest incidences of GSs and GBC, affecting predominantly women, since 2006, the Chilean government implemented a GBC prevention program, the Régimen General de Garantías Explícitas en Salud 26 (GES-26), aimed at increasing the number of cholecystectomies performed in high-risk individuals and thus hopefully reducing mortality from GBC [73,74]. The program guarantees abdominal ultrasound (US) with a short waiting list for individuals aged 35–49 years, and for the surgical removal of the gallbladder within 90 days in cases where GSs ≥ 3 cm or volume > 10 mL and/or a polyp ≥ 1 cm is detected. It further recommended that individuals at high risk of developing GBC undergo screening by abdominal US. This includes persons with a BMI ≥ 25, low educational level, at least one Mapuche surname (the Mapuche are the largest Native American group in Chile) and women with more than one child. Recent analyses suggested that such a targeted program may indeed be effective in reducing GBC mortality in selected populations of individuals at high risk of developing it [75,76]. This further promotes the conceptual strategy of a careful, tailored evaluation of the risk factors of each patient when considering the risk of GBC and the potential value of prophylactic cholecystectomy. In retrospect, given the patient’s advanced age but relatively good general conditions, the presence of DM, the high probability of GSs being present for an extended period, and the considerable quantity and dimensions of the stones present, a prophylactic cholecystectomy may have been a justifiable course of action, or at least an option to be evaluated.

**Table 2:** Selected patients at high risk who may potentially benefit from prophylactic cholecystectomy (chronological order). Legend: gallbladder carcinoma (GBC); gallstone (GS); prophylactic cholecystectomy (PC).

Author	Year	Conclusions
Kao et al. [45]	2005	PC after cardiac transplantation is the preferred management strategy for these patients with incidental GSs, as it reduces mortality and results in significant cost savings per quality-adjusted life-year.
Mohandas et al. [46]	2006	PC is recommended in populations with high incidence of GBC. In high-risk regions of India, PC should be offered to young healthy women as soon as they get diagnosed with asymptomatic GSs.
Behari et al. [47]	2012	Management of asymptomatic GSs should be selective cholecystectomy in high-risk subgroups.
Illige et al. [48]	2014	Whenever the benefits of operative treatment outweigh the risks of observation, PC should be offered to patients at high risk of biliary cancer or disease-related complications.
Mathur et al. [18]	2015	In India, GBC is the commonest GI cancer in women and 4th commonest cancer overall in the female population. Considering the epidemiology and clinical scenario of GBC, the current evidence today seems to justify a strategy of PC in GSs in North India.
Muroni et al. [49]	2015	PC should be considered for sickle cell disease patients with asymptomatic GSs, as it is safe and helps avoid emergency operations.
Lee et al. [50]	2022	It is clinically justifiable to advocate PC to minimize complication-related morbidity. For patients with high-risk profiles, including individuals with solitary large stones (>1–3 cm) or multiple small stones (<1) that would only increase over time; organ transplant recipients; those with red blood cell abnormalities (e.g., sickle cell anemia); concurrent gallbladder polyps; biliary sludge occupying > 50% of the gallbladder volume; old but still fit patients with a high stone burden.
Liu et al. [54]	2022	We are more in favor of PC as a standard strategy to treat GSs after gastric cancer surgery
Alves et al. [55]	2023	PC is recommended after cardiac transplantation and in patients with biliary microlithiasis and low preoperative surgical risk.
Fujita et al. [31]	2023	In patients at high risk for GBC (i.e., stones > 3 cm, polyps > 10 mm, porcelain gallbladder, thickened gallbladder walls, stone-filled gallbladders), cholecystectomy should be considered.

It has been demonstrated that persistent inflammation is associated with carcinogenesis through several mechanisms. These include cellular damage at the DNA level, the continual repair and regeneration of tissue, and the potential development of metaplastic and dysplastic changes from continuous attempts at restoring normal tissue [77]. Although cholelithiasis may not always cause acute cholecystitis and thus clinically evident signs and symptoms, it is a constant source of irritation and damage to the gallbladder tissues, creating a potentially conducive environment for malignant transformation over the course of years [21,78,79]. Furthermore, prolonged exposure to certain luminal factors, including elevated cholesterol and cytotoxic bile salts, leads to additional chronic damage to mucosa and lamina propria, as well as gallbladder leiomyopathy, which is characterized by pathological contraction or relaxation of smooth muscle tissue [80]. This chronic inflammatory microenvironment is also associated with tissue hypoxia, which results from compromised blood flow secondary to cycles of tissue damage and regeneration, ultimately causing also fibrosis and vascular remodeling. Furthermore, the presence of many large GSs results in the chronic dilation and stretch of the gallbladder wall, thereby compromising the parietal blood flow. A characteristic feature of numerous solid tumors is a hypoxic environment that has been linked to chemotherapy resistance, invasiveness, and metastasis [81]. The hypoxic conditions within the gallbladder wall, where healthy tissues are subjected to chronic inflammatory noxae deriving from long-standing cholelithiasis, facilitate the selection of cancer stem cells and the acquisition of genetic alterations that promote tumor progression, activating several molecules and signaling pathways [82]. CXCR4 is a key player in cancer biology, regulating crucial biological processes [9,83]. In hypoxic microenvironmental conditions, malignant cells upregulate the expression of CXCR4, enhancing tumor growth, leukocyte migration, angiogenesis, and consequently, metastatic potential. The elevated expression of CXCR4 in GBC has been associated with increased tumor aggressiveness and risk of recurrence, as well as a poor prognosis, highlighting its significance in the disease’s progression and its potential as a therapeutic target [84,85]. Similarly, the overexpression of CXCR3 and CXCR7 has been identified as an independent risk factor for a poorer prognosis in patients with GBC [86,87]. Hypoxia-inducible factors, upregulated under inflammatory conditions, represent another crucial element in cancer biology [88,89]. The survival rate for patients with HIF-1α positive staining has been reported as significantly lower than that for patients with HIF-1α negative staining, due to higher rates of lymph node metastases and venous invasion [81,90]. It has been demonstrated that hyperglycemia is associated with more aggressive form of certain cancers and higher level of HIF-1α expression [68].

It is noteworthy that, in contrast to the typical epidemiology of GBC, our patient exhibited the presence of SCC within the malignant mass. Squamous cell carcinoma of the gallbladder is a particularly uncommon occurrence, representing only approximately 1–4% of all malignant gallbladder tumors [91,92]. Conversely, adenocarcinoma (ADC) accounts for almost all cases of gallbladder malignancy [17,93]. When compared to ADC, SCCs often present as larger lesions and are more commonly associated with older age at presentation, higher histologic grades, and more advanced pathological stages [94]. Therefore, it may be hypothesized that SCC is more likely to cause cholecystitis by obstructing the cystic duct. However, given the significant disparity between SCCs and gallbladder ADCs, with the latter accounting for almost all of gallbladder malignancies, the majority of cases of cholecystitis secondary to a GBC could be attributed to the presence of an ADC type of neoplasia. A recent retrospective study analysing patients diagnosed with primary GBC, spanning from the 90s to early 2020s, reported that a third of them were discovered during the workup for acute calculous cholecystitis, with others getting diagnosed following imaging for other indications while being asymptomatic, or with imaging for nonspecific abdominal pain [95]. A noteworthy finding was that the survival curve for those who underwent surgical treatment and were reported to have experienced intraoperative spillage exhibited no significant difference when compared to those who had no spillage. As previously discussed, long-standing GSs and chronic cholecystitis are common factors involved in the development of GBC incidentally discovered during cholecystectomy for calculous cholecystitis. Conversely, GBC discovered within the context of acalculous cholecystitis is an uncommon occurrence. There have been reports of causes of acalculous cholecystitis also associated with GBC, including gallbladder adenomyomatosis [96,97,98], and infections from certain strains of Salmonella [57,99,100,101]. Considering the rarity of SCC of the gallbladder, the literature concerning its histologic features has been so far limited. A study conducted in the 1980s suggested a tendency for differentiated ADC or adeno-squamous carcinoma to be characteristic of calculous carcinoma, with more locally aggressive poorly differentiated ADC being associated with acalculous cases [102]. Microscopically, SCC presents with diffuse keratinization including pearl formation and dyskeratotic cells more frequently than ADC, exhibiting also desmosomes, larger nuclei, and eosinophilic cytoplasm [93,103]. Once carcinoma has developed, the tumor, due to its intrinsic biology, is typically locally aggressive, with early invasion of adjacent structures, and lymph node and distant metastases are often present at diagnosis. A lymph node from abdominal station 8a in our patient demonstrated the presence of carcinoma within, exhibiting immunohistochemical staining reaction for AE1-AE3, which have been previously documented to positively react both in the primary tumor mass and in lymph nodal metastases derived from a squamous type of carcinoma [104,105,106]. Direct infiltration of the liver is the most common pattern of spread, occurring in more than half of metastatic GBC cases and representing a significant risk factor for survival [107,108]. The median survival of patients with GBC with and without liver metastasis has been reported to be 7 and 22 months, respectively [105]. In our case, the histopathological findings of chronic inflammation and fibrosis, associated with poor differentiation of carcinoma with squamocellular features, serve to illustrate the complexity of the development and subsequent diagnosis of GBC.

Gallbladder carcinoma presents a diagnostic challenge due to the nonspecific nature of its symptoms and the often late-stage presentation. Given the well-established link between GSs and malignancy, asymptomatic GBC may be discovered incidentally during the perioperative management of complicated acute calculous cholecystitis, as illustrated by the current case. In cases where surgery is not required urgently, the preoperative diagnosis of a probable malignancy could markedly enhance therapeutic planning. However, the preoperative diagnosis remains challenging in patients requiring emergency cholecystectomy, due to the restricted time frame and the potential unavailability of experienced radiologists. In the acute setting, signs of gallbladder perforation should prompt consideration of GBC, particularly in elderly patients with high-risk factors [109].

Ultrasonography is the initial imaging, with reported sensitivity and overall accuracy for locally advanced diseases of 85% and 80%, respectively [17]. Typical findings suggestive of GBC include focal wall thickening, intraluminal mass lesions, and irregular mucosal surfaces [94]. On the contrary, acute cholecystitis tends to show diffuse wall thickening [110]. Gallbladder carcinoma should always be considered in the differential diagnosis when evaluating elderly patients with acute cholecystitis, especially females and diabetic, with focal or irregular mural thickening or enhancement of wall [111]. However, US has limitations in differentiating between benign and malignant lesions and detecting early-stage GBC, particularly in the presence of GSs or acute inflammation. In acute cholecystitis, the biliary sludge may be responsible for the generation of twinkling artefacts at Doppler US. The differentiation of these artefacts from actual vascular flow can be beneficial in the exclusion of a previously undocumented gallbladder mass [112]. When the tumor is flat or a malignant polyp is sessile in the context of cholelithiasis, US may fail to identify the lesion [17]. Furthermore, the presence of purulent content or thickened bile may reduce the probability of identifying a concomitant malignant mass, thereby limiting the applicability of US in the diagnosis of GBC in the context of acute cholecystitis [111]. Additionally, US does not accurately identify the full extent of the disease and has a limited assessment of locoregional and distant lymph node metastases, abdominal viscera, and peritoneum [113].

Computer tomography and MRI are therefore generally needed to evaluate local extent, nodal disease, and metastatic disease [43]. Computed tomography plays a crucial role in GBC staging, with a reported accuracy of up to 84% in determining the T stage (local extent) [114]. Additionally, CT scan has an 85% accuracy in predicting resectability, with precise depiction of direct hepatic or vascular invasion, lymphadenopathy, and distant metastasis [115]. Findings suggestive of GBC include gallbladder wall thickening with loss of its physiological trilaminar layering, an intraluminal mass, and lymphadenopathy. A hypodense area in the pericholecystic liver parenchyma may indicate the spread of the neoplastic disease. The absence of trilaminar layering, in conjunction with invasion of the liver parenchyma, is regarded as a highly suggestive indicator of advanced GBC. The homogeneous thickening of the gallbladder wall and the maintenance of its physiological layers are indicative of an acute inflammatory condition [116,117]. The triple-layer enhancement pattern observed with CT scan has been reported as useful for ruling out the presence of concomitant malignancy in acute cholecystitis [111]. Conversely, a two-layer pattern comprising a strongly enhancing thick inner layer and a weakly enhancing or non-enhancing outer layer, or a one-layer pattern comprising a heterogeneously enhancing thick layer, significantly associates with GBC [118]. In acute cholecystitis, the gallbladder is typically larger in volume than in GBC patients. However, the degree of wall thickening is more pronounced in malignant cases [111]. Regional fat stranding commonly associates with acute cholecystitis [117]. Despite the high reliability of CT examinations, it may still be challenging to obtain a definitive diagnosis of GBC based on imaging alone when inflammatory changes resulting from acute biliary pathologies are present. In the case of our patient, there were simultaneously signs suggestive of an acute inflammatory episode (i.e., enlarged gallbladder volume, diffuse wall thickening, regional fat stranding) and signs indicating a potential underlying malignancy (i.e., loss of physiological parietal layering, hypodense area in the pericholecystic liver parenchyma). Furthermore, the diffuse pattern of the malignancy presented a greater challenge to the preoperative diagnosis than would have been the case with a mass. Although MRI offers superior soft-tissue characterization of gallbladder and biliary tree than CT, its role in the management of acutely ill patients remains limited nowadays.

Surgery remains the cornerstone of treatment for resectable GBC [119], regardless of the histological type [120,121]. Radical cholecystectomy, which involves the removal of the gallbladder, a portion of the liver, and regional lymph nodes, is considered the standard surgical approach for early-stage tumors, but the overall prognosis remains unfavorable, with a high recurrence rate [122]. Adjuvant chemotherapy plays an important role and is suggested in patients with T ≥ 2 and/or node-positive disease, as in the case of our patient [26].

Tumor markers CA 19-9 and CEA, among many others, are routinely employed in the management of GBC. The initial measurement yielded values of 70.1 U/mL and 1.3 ng/mL, respectively. Despite surgery and adjuvant chemotherapy, the two markers increased gradually over time and were associated with the development of secondary lesions within the abdomen, as reported by CT scans during FU. Sachan et al. identified a statistically significant threshold in the survival of patients with initial CA 19-9 and CEA values of >37 vs. ≤37 U/mL and >4 vs. ≤4 ng/mL, respectively. The overall five-year survival rate for patients with CEA levels of ≤ 4 ng/mL and > 4 ng/mL was 42.8% and 12.5%, respectively. Patients with CA 19-9 ≤ 37 U/mL exhibited a five-year survival rate of 40%. In contrast, none of the patients with CA 19-9 > 37 U/mL survived for more than five years. Furthermore, CA 19-9 demonstrated higher sensitivity and specificity (52% and 80%, respectively) compared to CEA (51% and 72%, respectively) for the prediction of tumor burden in patients with GBC [123]. Similarly, Agarwal et al. reported a four-year survival rate of 78% in patients with GBC who underwent extended cholecystectomy when CA 19-9 was < 20 U/mL, compared to a 33% survival rate when CA 19-9 was > 20 U/mL [124]. Wen et al. reported that the simultaneous presence of elevated CEA (>5 ng/L) and CA 19-9 (>37 IU/mL) was an independent predictor of a poor prognosis in GBC patients undergoing resection [125]. In a study by Agrawal et al., patients with high serum CA 19-9 exhibited significantly lower median survival rates than patients with normal serum CA 19-9, but on the other hand, no significant differences were observed regarding the levels of CEA [126]. In a recent study, Sinha and colleagues reported that although elevated levels of any marker were not associated with survival, a significant reduction in tumor markers, including CA 19-9 and CEA, at three and six months post-surgery may be indicative of a response to treatment [127].

There is currently no international consensus on the optimal FU for these patients. In the initial two-year period, it is recommended that patients undergo regular FU visits at intervals of three to six months. These visits should include comprehensive clinical and laboratory assessments, with the addition of tumor marker analysis and a CT scan of the thorax, abdomen, and pelvis [128]. Subsequently, the frequency of visits could be extended to a period of six months, and eventually to a yearly appointment after a period of five years.

The continuum from clinically asymptomatic cholelithiasis to GBC through persistent subclinical localized inflammation represents a significant clinical challenge. This emphasizes the need for timely diagnosis and intervention in patients with GS disease to prevent acute surgical and long-term oncological complications. It is of the utmost importance to pay close attention to the identification of suspicious gallbladder lesions and masses during preoperative imaging in patients at high risk of malignancy who have been admitted for acute cholecystitis, given the increased diagnostic difficulty posed by the inflammatory context. Future analyses of large, targeted populations may assist in resolving the ongoing debate regarding the potential benefits of prophylactic cholecystectomy in patients with specific risk factors for surgical and oncological complications. Furthermore, it is of great importance to gain a deeper understanding of the relationship between long-standing GSs, chronic inflammatory states, gallbladder wall hypoxia, and the development of carcinoma. This knowledge will be invaluable in the development of effective preventive and therapeutic strategies for GBC. Targeting hypoxia-related pathways, such as CXCR4 and HIF signaling, may offer promising avenues for intervention in GBC. Nevertheless, further research is required to elucidate the molecular mechanisms underlying hypoxia-mediated carcinogenesis in the context of chronic cholecystitis.

## 4. Conclusions

In conclusion, our case illustrates some of the potential surgical and oncological complications of long-standing GSs and the diagnostic challenges of GBC, emphasizing the importance of considering neoplastic disease in high-risk patients with complex acute presentations of gallbladder pathology. A post hoc analysis of our patient, considering his general clinical picture, raises even more the question as to whether prophylactic cholecystectomy should be considered prior to the onset of biliary symptoms in selected high-risk patients. We argue that the choice to pursue a prophylactic cholecystectomy may have been justifiable in this patient. Some potential risk factors for the development of GBC in cholelithiasis patients may have been identified, but further research into the molecular mechanisms underlying this disease may offer insights into more effective preventive, diagnostic, and therapeutic strategies.

## Data Availability

Patients’ data registry of Azienda Ospedaliero-universitaria Senese di Santa Maria alle Scotte, Siena, Italy.
